# Chicken egg yolk antibodies (IgY) as non-antibiotic production enhancers for use in swine production: a review

**DOI:** 10.1186/s40104-015-0038-8

**Published:** 2015-08-25

**Authors:** Xiaoyu Li, Lili Wang, Yuhong Zhen, Shuying Li, Yongping Xu

**Affiliations:** School of Life Science and Technology, Dalian University of Technology, Dalian, 116024 China; Ministry of Education Center for Food Safety of Animal Origin, Dalian, 116620 China; Department of Pharmacy, Dalian Medical University, Dalian, 116044 China; Dalian SEM Bio-Engineering Technology Co. Ltd, Dalian, 116620 China

**Keywords:** Diarrhea, Disease control, Egg yolk antibodies, IgY, Swine

## Abstract

In recent years, the use of in-feed antibiotics for growth and disease prevention in livestock production has been under severe scrutiny. The use and misuse of in-feed antibiotics has led to problems with drug residues in animal products and increased bacterial resistance. Chicken egg yolk antibodies (IgY) have attracted considerable attention as an alternative to antibiotics to maintain swine health and performance. Oral administration of IgY possesses many advantages over mammalian IgG such as cost-effectiveness, convenience and high yield. This review presents an overview of the potential to use IgY immunotherapy for the prevention and treatment of swine diarrhea diseases and speculates on the future of IgY technology. Included are a review of the potential applications of IgY in the control of enteric infections of either bacterial or viral origin such as enterotoxigenic *Escherichia coli*, *Salmonella* spp., rotavirus, porcine transmissible gastroenteritis virus, and porcine epidemic diarrhea virus. Some potential obstacles to the adoption of IgY technology are also discussed.

## Introduction

Antibiotics have been widely used in the swine industry for more than 50 years. The efficacy of antibiotics in increasing growth rate, improving feed utilization, and reducing incidence of disease is well documented [1]. In general, sub-therapeutic levels of antibiotics in swine diets increase the growth rate by an average of 16 % in weanling pigs, 11 % in growing pigs, and 4 % in growing-finishing pigs [1]. In addition, antibiotics are also used for disease prevention (prophylactic doses) and treatment (therapeutic doses). However, serious concerns have arisen with regard to the potential risks for human health including drug residues in meat products and increased bacterial resistance due to the use and misuse of in-feed antibiotics [2]. As a result, the European Union has totally banned the use of antibiotics for growth promotion since January 2006 [3], while the US Food and Administration (FDA) has been phasing out non-medical antibiotic use for livestock since December 2013 [4].

Diarrheal disease is a frequent cause of heavy economic losses for swine producers. A major challenge currently facing the swine industry is to develop alternative means for controlling diarrhea in young pigs (particularly neonatal and early-weaned piglets) that are not only cost effective, but also allow for sustainable pork production. In the past two decades, a variety of materials have been tested as effective alternatives to antibiotics to maintain swine health and performance. The most widely researched alternatives include enzymes [5], organic acids [6], pro- and prebiotics [7–9], herbal extracts [10, 11] and neutraceuticals such as copper and zinc [12, 13]. However, these alternatives produce limited growth promotion and protection against pathogens.

Recently, egg yolk antibodies, generally referred to as IgY, have attracted considerable interest as an alternative to antibiotics for growth promotion in the presence of disease causing organisms [14]. IgY possesses a large number of advantages over mammalian IgG such as cost-effectiveness, convenience, and high yield [15]. Oral administration of specific IgY antibodies has been shown to be highly effective against a variety of intestinal pathogens which cause diarrhea in animals and human such as enterotoxigenic *Escherichia coli* (ETEC), *Salmonella* spp.*,* bovine and human rotaviruses, bovine coronavirus, porcine transmissible gastroenteritis virus (TGEV), and porcine epidemic diarrhea virus (PEDV) [14, 16].

Following a brief description of IgY technology and the advantages of IgY, this review will focus on the potential to use specific IgY as a production enhancer in swine production, and discuss the potential obstacles to the adoption of IgY technology.

## IgY technology

As described more than 100 years ago, Klemperer [17] first demonstrated that avian maternal antibodies are transferred from serum to egg yolk in order to protect the developing embryo from potential pathogens but at that time there was no scientific application for this knowledge. However, when animal welfare became a matter of serious ethical concern for the scientific community, the results of Klemperer began to receive more interest, particularly since the 1980s.

Since 1996, IgY technology (i.e. the production and use of IgY) has become an internationally accepted practice [18]. In 1996, the European Centre for the Validation of Alternative Methods (ECVAM) workshop strongly recommended that IgY should be used as an alternative to mammalian antibodies [19]. In 1999, IgY technology was approved as an alternative method for supporting animal welfare by the Veterinary Office of the Swiss Government (Office Vétérinaire Fédéral).

Details concerning the production of specific IgY have been reviewed by Schade et al. (2005) [20]. Briefly, in order to produce specific IgY antibodies, laying hens are immunized with specific foreign pathogens which induce immune responses, including the production of antibodies with activity against these specific disease conditions. These antibodies are then transferred to the egg yolk and deposited in large quantities. Booster immunizations are usually given to ensure continued transfer of antibodies from the hen’s serum to the egg yolk. These antibodies are then extracted from the egg yolk and processed to be administered directly to the animal or incorporated into diets.

Antibodies can be administered in several forms including whole egg powder, whole yolk powder, a water-soluble fraction powder or purified IgY. Antibody production and titer development as a result of immunization are not very predictable. Variables influencing immunization include the antigen (dose, MW), type of adjuvant used, route of immunization, immunization frequency and the chicken itself (such as housing conditions, age, breed, egg laying capacity).

## Advantages of IgY

The use of chickens as the immunization host for antibody production provide a number of advantages over production methods using mammals. The most significant advantage is that egg collection is non-invasive compared with the stressful bleeding of animals to obtain serum. In addition, the high and long-lasting titer produced in chickens reduces the need for frequent booster injections. Another advantage is that the production of IgY antibodies against highly conserved mammalian proteins is more successful in chickens than in other mammals [21] and requires much less antigen to induce an efficient immune response due to the phylogenetic distance between chickens and mammals [22].

A hen can be considered as a small "factory" for antibody production. One hen can produce more than 22.5 g of total IgY per year of which 2 to 10 % are specific antibodies [14]. This extraordinary quantity is equivalent to the production of 4.3 rabbits over the course of a year. The cost for maintaining laying hens are also lower than those for mammals such as rabbits [20]. Therefore, egg yolk offers a more hygienic, cost-efficient, convenient and rich source of antibodies compared with traditional production methods using mammals. In contrast to antibiotics, the use of IgY is environmentally-friendly and elicits no undesirable side effects, disease resistance or toxic residues [23].

In terms of function, unlike mammalian IgG, IgY does not activate mammalian complement and also does not interact with mammalian Fc and complement receptors. As well, IgY does not bind to protein A, protein G or rheumatoid factor [15]. These differences provide great advantages for the successful application of IgY technology in many research areas, diagnostics [24], antibiotic-alternative therapy [15] and xenotransplantation [25].

## Mode of action

**Fig. 1:**
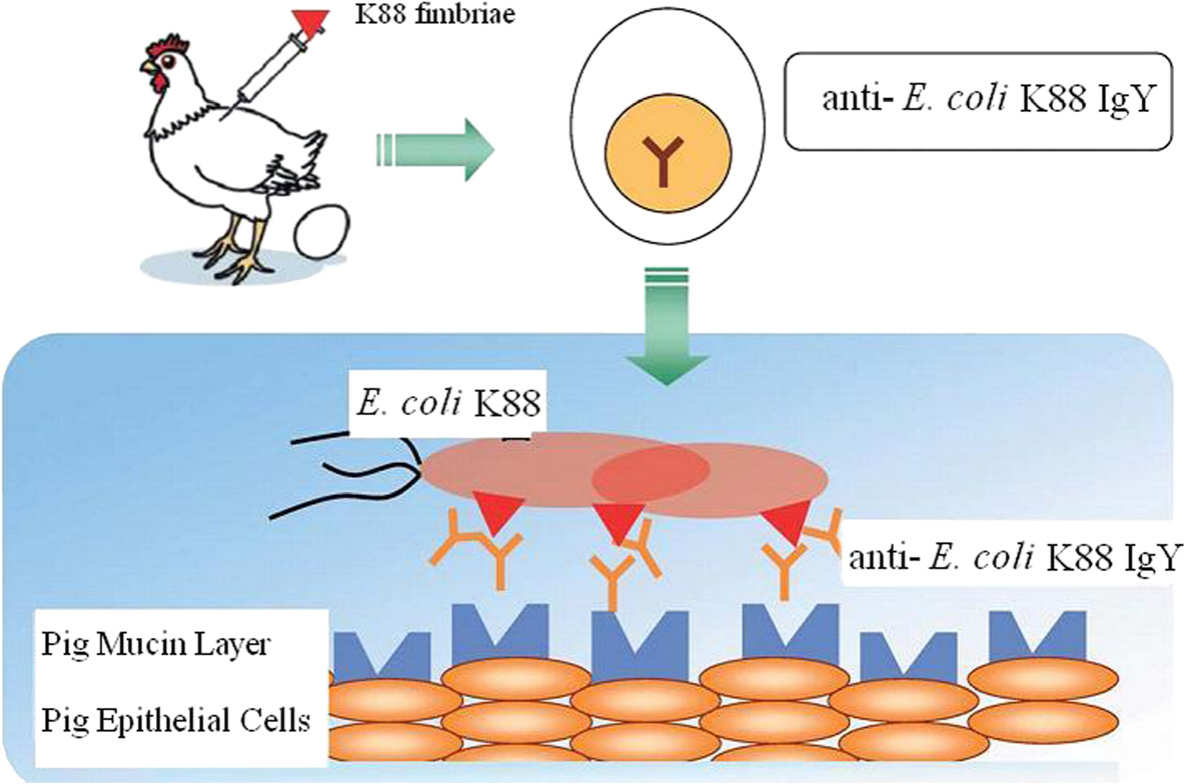
Possible primary mode of action by which IgY protects pigs against *E coli* K88 induced diarrhea. Modified from Hatta et al. [85]

The exact mechanisms through which IgY counteracts pathogen activity have not been determined. However, several mechanisms have been proposed by Xu et al. (2011) [14] including agglutination of bacteria, inhibition of adhesion, as well as opsonization followed by phagocytosis and toxin neutralization. Among these, inhibition of adhesion is considered the primary mechanism by which specific IgY functions. Briefly, IgY antibodies generated against intestinal disease causing organisms may reduce the incidence of disease by preventing the attachment of pathogens to the intestine, such as blocking the mucosal receptor, interfering with binding to mucins, or neutralizing the colonization factor (such as the outer membrane protein, lipopolysaccharide, fimbriae (or pili), and flagella) [26]. The possible primary mode of action by which IgY protects pigs against *E coli* K88 induced diarrhea is illustrated in Fig. 1.

## IgY applications in swine production

Diarrhoea in neonatal and post-weaning pigs can be caused by a number of causative agents and has become a serious problem in the swine industry due to the trend towards large intensive herds and early weaning (i.e. 14–21 rather than 21–28 days of age). The potential applications for using orally administered IgY in the control of enteric infections of either bacterial or viral origin in piglets have been studied at length and are summarized in Tables 1 and 2.

**Table 1:** Effect of IgY against diarrhea caused by bacterial pathogens in piglets

Items			Mortality, %		Outcome measure considered-Mortality (M) or Diarrhea (D)
			Intervention	Control	
Prophylactic effect					
Ref.	Pathogens	Piglet age			
Imberechts et al. [33]	F18+ ETEC	Weaned (21–28 d)	33	66	D
	F18+ ETEC	Weaned (21–28 d)	25	75	D
	F18+ ETEC	Weaned (21–28 d)	0	25	M
Marquardt et al. [34]	K88+ ETEC	Neonatal (3-day-old)	12.5	62.5	M
	K88+ ETEC	Weaned (21-day-old)	0	30	M
	K88+ ETEC	Weaning (14-18-days-old)	1.9	3.9	D
Owsu-Asiedu et al. [36]	K88+, F18+, F41+ ETEC	Early-weaned (10-day-old)	30	73	D
	ETEC K88+, F18, F41	Early-weaned (10-day-old)	33	100	D
Owsu-Asiedu et al. [37]	K88 + ETEC	Early-weaned (10 ± 1 days old)	6.6	40	M
Owsu-Asiedu et al. [38]	K88 + ETEC	Early-weaned (10-day-old)	0	33	M
Chernysheva et al. [76]	K88 + ETEC	Newly weaned (about 22-day-old)	66	58.3	D
Chu et al. [86]	K88 + ETEC	Neonatal (3-day-old)	16.7	66.7	M
Li et al. [83]	K88 + ETEC	Weaned (40-day-old)	0	75	D
Therapeutic effect					
Ref.	Pathogens	Piglet age			
Yokoyama et al. [27]	K88 + ETEC	Newborn	0	86	M
	K99 + ETEC	Newborn	0	100	M
	987P + ETEC	Newborn	0	80	M
Yokoyama et al. [87]	F18+ ETEC	Weaned (28-day-old)	0	11	D
Yang et al. [88]	K88 + ETEC	Newborn	0	85.7	M
	K99 + ETEC	Newborn	0	100	M
	987P + ETEC	Newborn	0	80	M
Xu et al. [89]	K88 + ETEC	Newborn	0	33.3	M
	K99 + ETEC	Newborn	0	66.7	M
	987P + ETEC	Newborn	0	50	M
Chu et al. [86]	K88 + ETEC	Weaned (21-day-old)	0	25	M

**Table 2:** Effect of IgY against diarrhea caused by viral pathogens in piglets

Items			Mortality, %		Outcome measure considered-Mortality (M) or Diarrhea (D)
			Intervention	Control	
Prophylactic effect					
Ref.	Pathogens	Piglet age			
Kweon et al. [66]	PEDV	Neonatal (3-day-old)	26	58	M
	PEDV	Neonatal (3-day-old)	41	71	M
	PEDV	Neonatal (3-day-old)	50	66	M
Zuo et al. [61]	TGEV	Newborn	12.5	57	M
Vega et al. [73]	Human Rotavirus	Neonatal (1-day-old)	0	100	D
Therapeutic effect					
Ref.	Pathogens	Piglet age			
Song et al. [67]	PEDV	Neonatal (1-day-old)	16.7	100	M
Cui et al. [62]	TGEV, PEDV	Neonatal (1-day-old)	0	100	M

## Antimicrobial and performance effects

### Enterotoxigenic *Escherichia coli*

Diarrhea due to enterotoxigenic *Escherichia coli* (ETEC) is by far the most common enteric colibacillosis encountered in neonatal and post-weaned pigs [27–29]. ETEC can cause diarrhea in piglets by colonization in the small intestine and thereafter through the production of either one or two enterotoxins namely heat-labile enterotoxin (LT) and heat-stable enterotoxins (ST), which induce massive fluid and electrolyte secretion into the gut lumen [30].

The strains of *E. coli* associated with intestinal colonization which cause severe diarrhea are those expressing the K88 (or F4), K99 (or F5), 987P (or F6), F18 and F41 fimbrial adhesions. These fimbrial adhesions mediate the adhesion of *E. coli* to the epithelial mucosa lining of the intestine and thereby contribute to their virulence. Among the different ETEC, those expressing the K88+ fimbrial antigen are the most prevalent forms causing *E. coli* infection world-wide [31]. It has been estimated that K88 + ETEC are responsible for more than half of the piglet mortality which occurs annually [32].

Oral administration of IgY offers a potential prophylactic and therapeutic approach for controlling *E. coli*-induced diarrhea in piglets. IgY has been shown to successfully control intestinal infections of *E. coli* K88, K99, 987P, and F18 in young pigs [27, 33, 34]. Yokoyama et al. [27] showed that orally administered crude IgY (the water-soluble fraction) generated against *E. coli* K88, K99, or 987P fimbriaes was protective against infection from three homologous strains of *E. coli* in a dose-dependent manner in colostrum-deprived piglets. *In vitro*, *E. coli* K88, K99, and 987P strains adhered equally to porcine epithelial cells from the duodenum and ileum but failed to so in the presence of homologous anti-fimbrial IgY [27].

The group of Marquardt [26, 34] from the University of Manitoba in Canada carried out some excellent studies on the passive protective effects of IgY against ETEC K88 fimbriae in neonatal and early-weaned piglets. In an animal feeding study, 21-day old pigs were orally challenged with high doses of ETEC K88 at 0 and 5 h of the experiment. They were then oral administered with crude IgY (the water-soluble fraction) three times a day for two consecutive days after the first *E. coli* challenge. Control piglets (treated with IgY from non-immunized hens) had severe diarrhea within 12 h and lost weight and 30 % of the pigs died within 48 h of infection. In contrast, the pigs given IgY from immunized hens exhibited no signs of diarrhea 24 and 48 h after treatment, and had a positive weight gain. Furthermore, this group performed studies on the practical use of IgY in a field trial. Their experiment showed suppression of the incidence and severity of diarrhea in 14-18-day-old weaned piglets fed specific IgY powder, which were much lower than those fed a commercial diet containing an antibiotic. The number of pigs in this study was not large and it would be desirable to repeat this study with a greater number of animals.

In another study with F18+ *E. coli*, it was shown that the F18 antibodies diminished the incidence of diarrhea and death in weaned piglets infected with F18+ *E. coli* [33]. Zuniga et al. [35] also reported that weaned pigs fed a basal feed plus 5 % (w/w) egg powder with IgY antibodies against the same fimbrial variant (F18ab or F18ac) were fully protected when the pigs were challenged with F18+ *E. coli*.

In addition to reducing the incidence and severity of piglet diarrhea, several studies have shown that IgY has growth promoting effects in early-weaned pigs, similar to spray-dried animal plasma and spray-dried porcine plasma [36–39]. Table 3 shows the results of an experiment where the performance of pigs fed specific anti-K88 antibodies was compared with that of pigs fed diets supplemented with zinc oxide, fumaric acid or antibiotics after oral ETEC K88 challenge [37]. All four feed additives successfully increased pig performance compared with unsupplemented pigs with significant reductions observed in scour score and piglet mortality. In this experiment, IgY was equal to antibiotics in enhancing pig performance. Table 4 shows the effects of adding egg yolk containing specific anti-K88 antibodies (EGG), EGG-exchange, or spray dried porcine plasma to a corn-soybean meal-based (CON) diet on performance, plasma urea and weight of pancreas of weaned pigs that were not challenged with ETEC K88 challenge [39]. The results show that addition of EGG to the CON diets reduced plasma urea nitrogen concentration, and increased feed intake by 23 % and tended to increase weight gain by 28 % in phase II. This study indicates that inclusion of EGG in the diets for pigs immediately after weaning can significantly affect their future growth performance. Unfortunately, limited numbers of pigs were used in this study and a repeat with greater numbers would certainly be welcomed.

**Table 3:** Effect of IgY, zinc oxide, fumaric acid and antibiotics on the performance and intestinal morphology of 10 to 24 day old pigs fed diets based on pea protein concentrate

Items	Control	IgY	Zinc oxide	Fumaric acid	Carbadox	SEM
Weight gain, g/d	100.9	151.2	158.9	155.4	152.6	16.6
Feed intake, g/d	141.0	208.1	214.7	211.6	222.4	15.3
Feed conversion	1.39	1.38	1.35	1.36	1.45	0.04
Scour score	2.7	1.3	1.4	1.3	1.1	-
Mortality, %	40.0	6.6	13.3	6.6	13.3	-
Villus height, m	355	564	488	573	570	20.0
Crypt depth, m	204	183	190	207	204	10.1
Villous height/crypt depth	1.7	3.1	2.6	2.8	2.8	0.11

Owusu-Asiedu et al. [37]

**Table 4:** Effect of EGG, EGG-X, or SDPP on performance, plasma urea and pancreas weight of weaned pigs fed diets based on corn-soybean meal^1^

Items	Control	EGG	EGG-X	SDPP	SEM
Weight gain, g/d					
d 0-14	124^b^	151^ab^	128^b^	185^a^	12.4
d 14-25	332	425	349	398	25.9
Feed intake, g/d					
d 0-14	191	219	197	222	12.3
d 14-25	530^b^	655^a^	583^ab^	610^ab^	37.4
Feed conversion					
d 0-14	1.43	1.43	1.47	1.18	0.067
d 14-25	1.54	1.54	1.67	1.54	0.033
Plasma urea N, mmol/L					
d 0-14	3.8	3.9	4.3	3.7	0.34
d 14-25	3.4^a^	3.0^b^	3.6^a^	2.6^b^	0.22
Pancreas, g/kg of body weight	1.07^b^	1.22^ab^	1.34^a^	1.08^b^	0.071

Heo et al. [39]

^1^EGG, egg yolk obtained from hens immunized with ETEC K88 antigens; EGG-X, 650.0 g/kg normal egg and 350.0 g/kg inulin; SDPP, spray dried porcine plasma

^ab^Means in a row followed by same or no letter do not differ (*P >* 0.05)

Although adhesin-mediated colonization is a precondition for ETEC pathogenesis, enterotoxins are thought to be the central virulence determinants leading to diarrhea diseases and may also play a role in the colonization process [30, 40, 41]. Therefore, the ideal protective agent against ETEC infection should include protection against both adhesin antigens and enterotoxins [30]. In contrast with LTB and STa, enterotoxins STa and STb are poorly immunogenic because of their small size, but they can attain immunogenicity when coupled chemically or genetically to an appropriate carrier [42, 43]. In order to make useful toxoids, we constructed polyvalent enterotoxin STa-LTB-STb DNA and protein vaccines endowing immunogenicity to both STa and STb [44]. Laying hens were immunized with DNA vaccines and obtained antitoxic antibodies from egg yolks and this was confirmed by indirect ELISA. The polyvalent DNA vaccine pCI-STa-LTB-STb expressed the STa-LTB-STb fusion peptide *in vitro* in cultured Hela cells. These egg yolk antibodies were able to neutralize the natural toxicity of STa and LTb with the highest dilution of 1/2 and 1/32 in a suckling mouse assay [45]. These results indicate that the recombinant STa-LTB-STb protein has the potential to serve as an effective and convenient polyvalent toxoid which can provide broad protection against ETEC-induced diarrhea. This study was conducted with mice and a piglet study should be conducted to confirm these findings.

### Salmonella

*Salmonella* infection has been recognized as one of the most common foodborne diseases in humans, causing 1.4 million cases with an estimated economic impact of $2.4 billion each year in the United States [46, 47]. This disease can occur via foodborne transmission, animal contact, or environmental spread [48, 49], and farm animals are the most likely source of human salmonellosis [50, 51]. Therefore, it is important to control this disease, not only to reduce productive losses in domestic livestock, but also to prevent its transmission into the human food chain. Salmonella species, *S. enteritidis* and *S. typhimurium*, in particular, are thought to be the major agents of human salmonellosis [46]. They are non-host serotypes which can cause disease syndromes like gastroenteritis and systemic infectious in a wide range of animal species, including humans.

Studies by Yokoyama et al. [52] investigated the efficacy of IgY antibodies specific for outer membrane protein (OMP), lipopolysaccharide (LPS), or flagella (Fla) for controlling *S. typhimurium* or *S. enteritidis*. They treated mice orally with an appropriate placebo or purified IgY following a challenge with *S. enteritidis*. Antibody treatment resulted in survival rates of 80, 47 and 60 % using OMP-, LPS-, and Fla-specific antibodies, in contrast to survival rates of only 20 % in control mice. In the *S. typhimurium* trial, the survival rate was 40, 30 and 20 % using OMP-, LPS-, or Fla-specific antibodies, in contrast to 0 % in the control mice. These preliminary results suggest that antibodies against specific *Salmonella* proteins can control salmonellosis when orally administrated to mice.

Studies with pigs have been reported [53, 54]. Unfortunately, reports where similar benefits of IgY to those found in mice were not obtained with pigs. Mathew et al. [53] found that feeding egg yolk powder containing anti-*Salmonella* IgY antibodies may not be particularly effective in reducing *Salmonella* shedding in pigs. In this experiment, specific egg yolk powder derived from chickens challenged with purified *Salmonella typhimurium* antigens (fimbrial protein, OMP, and LPS) was included in swine feed. Treatments were provided beginning on day 3 of the experiment, and all pigs were challenged with *Salmonella typhimurium* on day 7. Fecal samples were collected on days 0, 7, 8, 12, 14, 21, 58, 88, and 118 to determine shedding of *Salmonella*. The results showed that in-feed IgY antibodies did not diminish the shedding of *Salmonella*, and was inferior to antibiotic treatment. The failure of IgY to improve performance is suspected to be due to the fact that the invasive nature of *Salmonella* allowed the organism to by-pass the gut and the dietary treatments by moving through vascular and/or lymphatic routes directly into the colon [55–57]. However, available data are still too limited to allow reliable conclusions regarding the possible efficacy of IgY to control *Salmonella* infection in swine.

## Antiviral and performance effects

### Porcine transmissible gastroenteritis virus

Porcine transmissible gastroenteritis virus (TGEV), a porcine coronavirus, can cause a highly contagious enteric infection in swine of all ages [58]. The infected piglets develop significant clinical signs, including vomiting, emaciation and severe diarrhea. The disease is especially severe in the animals less than 2 weeks old with a mortality of nearly 100 % [59, 60]. With the lack of successful vaccines to prevent a TGE outbreak, the disease occurs frequently in swine farms. It has been shown that specific IgY has great potential as an alternative prophylactic approach like colostral antibodies against TGEV [61, 62]. In a prophylactic efficacy experiment, oral administration with IgY significantly increased newborn piglet survival rate (87.5 %) after challenge exposure compared with the control (43 %), whereas the therapeutic effects demonstrate that mortality was dramatically reduced by orally administered IgY in two farms that showed TGEV positive results [61]. Unfortunately, piglet performance was not monitored in this study.

### Porcine epidemic diarrhea virus

Porcine epidemic diarrhea virus (PEDV) is another important enteric viral pathogen that is responsible for neonatal piglet diarrhea [63, 64]. Although the clinical symptoms of PEDV infection are similar to TGEV infection, PEDV is antigenically different from TGEV. Epidemiological observations have indicated that the spread of disease seems to be slower, but rather persistent compared with a TGEV outbreak [65]. It has been shown that IgY can be an alternative method for conferring protection in piglets against PEDV [62, 66, 67]. IgY was found to reduce mortality in piglets after challenge exposures. The field application of IgY from three farms also revealed the survival rate was increased significantly in pigs treated with IgY (49.24 %) compared with a control group (33.71 %).

### Rotavirus

Rotavirus is a major pathogen of infectious gastroenteritis, not only in children and infants, but also in domestic animals. In humans alone, it has been estimated that rotavirus infections result in several million deaths each year [68]. Animals are also seriously affected by this virus. Rotavirus from calves, pigs, mice, foals, infant humans, lambs, chickens, and turkey are antigenically related [69]. Therefore, the appropriate anti-rotavirus antibodies will react with the virus present in any of these species.

IgY has been shown to successfully control intestinal infection of rotavirus in newborn calves and mice [70–73]. Studies with pigs have been reported and similar positive results were obtained [74]. A passive treatment based on human rotavirus specific IgY antibodies not only prevented gnotobiotic piglets from developing diarrhea caused by the prevalent strain of Wa G1P [8] HRV, but also significantly reduced the amount of infectious virus shed compared with a negative control group.

## Current challenges

There are many obstacles that can limit the use of IgY for the control of diarrhea diseases in swine. The most important issues which need to be addressed are as follows.

### The stability of IgY in the gastrointestinal tract when they are fed to swine

Although the beneficial effects of pathogen-specific IgY in animals have been known for about 20 years, results on the experimental application of these antibodies in swine have not always been consistent. There are several reports where IgY failed to improve pig performance [75, 76]. The most likely explanation for the failure of the treatment is that the antibody failed to survive passage through the gastrointestinal tract as a result of its susceptibility to proteolysis [77]. IgY, being a glycoprotein, is sensitive to the same denaturing conditions as most proteins. It has been reported that IgY is fairly resistant to digestion by intestinal proteases, but the activity of IgY was decreased at pH 3.5 or lower and almost completely lost with irreversible change at pH 3 [78]. In addition, the inactivation at low pH is further enhanced by the presence of pepsin. In contrast to very young pigs, the stomach of older pigs has a low pH and pepsin, and therefore IgY may not be effective in controlling post-weaning *E. coli* diarrhea in older animals.

Yokoyama et al. [79], in an excellent study, detected the rate of IgY passage through the gastrointestinal tract of pigs and its ability to retain its activity in the different sections of the tract. Their results demonstrated that IgY is readily absorbed within 24 h by the newly born pig. IgY has a serum half-life of 1.85 days in newborn pigs, which is shorter than the reported serum half-life of 12 to 14 days for homologous IgG (colostral antibodies). The amount of IgY absorbed into the circulation when administered in pigs decreased with increasing age of pigs.

Since the primary target site of IgY is the small intestine, in order for it to function, it must be able to survive passage through the harsh environmental conditions found in the stomach. Microencapsulation techniques have been developed to protect IgY from gastric inactivation [77, 80–83]. Table 5 shows the results of an experiment where chitosan-alginate microcapsules were used for oral delivery of egg yolk immunoglobulin in weaned pigs challenged with enterotoxigenic *E. coli* K88 [83]. The percentage of pigs with diarrhea 24 h after treatment and the diarrhea score were improved in pigs receiving encapsulated IgY compared with non-encapsulated IgY. In addition, weight gain over the three day period was significantly higher in pigs receiving encapsulated IgY compared with non-encapsulated IgY. Both encapsulated and non-encapsulated IgY treatments were numerically superior to an aureomycin treated group. However, this process will undoubtedly add additional costs. Additional study will be needed before microencapsulation can be applied under commercial conditions.

**Table 5:** Effect of encapsulated IgY on performance and the incidence of diarrhea in pigs challenged with K88+ ETEC^1^

Items	No. of pigs	Percentage of pigs with diarrhea after specific times (Fecal score in brackets^2^)				Weight gain, g^3^	Recovery rate, (%)
		9 h	24 h	48 h	72 h		
Negative control, unchallenged	4	0 % (0.5)	0 % (0)	0 % (0.4)	0 % (0)	+1400 ± 129^a^	——
Positive control	4	75 % (2.5)	75 % (2.5)	75 % (2.0)	75 % (2.0)	−162.5 ± 25^d^	0 %
Non-encapsulated IgY	4	100 % (2.0)	75 % (1.3)	25 % (1.0)	0 % (0)	+937.5 ± 111^b^	100 %
Microencapsulated IgY	4	75 % (2.0)	0 % (0)	0 % (0)	0 % (0)	+1325 ± 119^a^	100 %
Aureomycin	4	100 % (2.0)	50 % (2.0)	75 % (1.5)	50 % (1.5)	+650 ± 71^c^	50 %

Li et al. [83]

^1^All pigs except negative control were orally challenged with 5 mL of viable *E. coli* K88 organisms (10^11^ cfu/mL per pig) at time 0 h. Challenged pigs were left untreated (positive control) or treated three times (−1, 4 and 9 h after bacterial challenge) on the first day and twice a day for two consecutive days each time with 0.4 g non-encapsulated IgY, 2 g of microencapsulated IgY (equivalent to 0.4 g of IgY) or 0.25 g of aureomycin. Diarrhea and weight gain were assessed for 3 days after challenge

^2^FC score is the mean fecal consistency score: 0, normal; 1, soft feces; 2, mild diarrhea; 3, severe diarrhea. Pigs with a fecal score of < 1 were considered not to have diarrhea

^3^Means in a column followed by same or no letter do not differ (*P >* 0.05)

### The stability of IgY when subjected to feed processing

Egg-yolk antibodies can be administrated to young pigs either as a preventive or prophylactic treatment or as a therapeutic treatment after infection occurs. It appears that adding antibodies to swine feeds in the form of whole egg powder or whole yolk powder may be the most practical method of inclusion of IgY for preventive or prophylactic treatment. It is not known if IgY antibodies can tolerate heat-based feed processing techniques such as pelleting. Presumably the stability of IgY antibodies would be similar to that for enzymes and that stability would be greatly influenced by temperature, duration of heat treatment and cooling period as well as the moisture content of the diet. It is well known that a change in moisture content from 95 to 90 or 85 % dry matter can dramatically decrease the stability of proteins when heated to high temperatures. Preliminary studies by Marquardt et al. [84] have demonstrated that low temperature steam pelleting (70 °C) did not reduce antibody titer. Further studies need to be carried out on this problem

### Specificity of IgY

IgY is an attractive and effective alternative approach to antibiotics due to its high specificity. It should be noted that swine are exposed to many infectious agents in commercial operations and therefore the swine industry will benefit more from IgY antibodies if they are produced against a mixture of common disease causing organisms rather than one specific disease. If this approach works well, it may help to justify, to some extent, commercial application of these antibodies.

### Amount of product added to the diet or administrated by gavage

The efficacy of IgY in the gastrointestinal tract is related to two factors namely the dose of antibodies and the concentration of pathogen. The results of Yokoyama et al. [79] demonstrated that antibodies pass through the digestive system within a relatively short period of time (slightly more than 24 h). In addition, they can reach all sections of the gastrointestinal tract in less than 10 h. The supply of antibodies in the intestine must be sufficiently high to prevent binding of pathogen to the intestinal receptors. The antibody must also be continuously or nearly continuously available in the intestine. Studies by Marquardt et al. [34] showed that 1.5 g per day per piglet was sufficient to prevent diarrhea induced by infection with 10^10^ ETEC and the addition of 0.2 % of egg-yolk antibody in the diet was preventive against diarrhea in commercial farms. However, a considerable amount of additional research must be carried out to identify the best antigens to use and the appropriate prevention or treatment protocols for control of intestinal pathogens such as different strains of *E. coli*.

### The production cost of high quality IgY antibodies

At present, the production cost of high quality IgY antibodies is higher than the cost of routine antibiotic inclusion [84]. Therefore, the development of methods for large-scale production of IgY which produce a high recovery and purity of IgY are needed. The method should be simple, economical and require few chemicals. In addition, there is no consensus on the most suitable IgY extraction method for commercial application [20] and this requires further study.

## Conclusions

Oral administration of specific IgY appears to have considerable potential as a means of controlling diarrhea diseases and exerting growth-promoting activity in swine. IgY technology will probably provide the best alternative to antibiotics. Some advantages of IgY in the control of swine diseases are:

1. They are highly effective.
2. They are highly cost-effective with only a small amount of antibody required per pig.
3. Collection of eggs is non-invasive.
4. The treatment is safe and live organisms are not used.
5. The procedure is environmentally friendly.
6. No toxic residues are produced and there is no development of resistance.
7. The treatment can be used to control many different types of pathogens.
